# Newer generations of multi-target CAR and STAb-T immunotherapeutics: NEXT CART Consortium as a cooperative effort to overcome current limitations

**DOI:** 10.3389/fimmu.2024.1386856

**Published:** 2024-05-08

**Authors:** Beatriz Martín-Antonio, Belén Blanco, África González-Murillo, Laura Hidalgo, Jordi Minguillón, Gema Pérez-Chacón, Next Generation CART MAD Consortium, Jordi Minguillón

**Affiliations:** ^1^ Department of Experimental Hematology, Instituto de Investigación Sanitaria-Fundación Jiménez Diaz (IIS-FJD), Madrid, Spain; ^2^ Cancer Immunotherapy Unit (UNICA), Department of Immunology, Instituto de Investigación Sanitaria Hospital 12 de Octubre (imas12), Madrid, Spain; ^3^ Department of Pediatric Hematology and Oncology, Advanced Therapies Unit, Fundación Investigación Biomédica Hospital Infantil Universitario Niño Jesús, Madrid, Spain; ^4^ Cellular Biotechnology Unit, Instituto de Salud Carlos III (ISCIII), Madrid, Spain; ^5^ La Paz Hospital Institute for Health Research (IdiPAZ), Hospital Universitario La Paz. Universidad Autónoma de Madrid (UAM), Madrid, Spain; ^6^ Immunity, Immunopathology and Emergent Therapies Group. Instituto de Investigaciones Biomedicas Sols-Morreale. CSIC-UAM, Madrid, Spain

**Keywords:** CAR-T cells, NK cells, TILs, TCR therapy, immunological synapse, cancer

## Abstract

Adoptive T cellular immunotherapies have emerged as relevant approaches for treating cancer patients who have relapsed or become refractory (R/R) to traditional cancer treatments. Chimeric antigen receptor (CAR) T-cell therapy has improved survival in various hematological malignancies. However, significant limitations still impede the widespread adoption of these therapies in most cancers. To advance in this field, six research groups have created the “NEXT Generation CART MAD Consortium” (NEXT CART) in Madrid’s Community, which aims to develop novel cell-based immunotherapies for R/R and poor prognosis cancers. At NEXT CART, various basic and translational research groups and hospitals in Madrid concur to share and synergize their basic expertise in immunotherapy, gene therapy, and immunological synapse, and clinical expertise in pediatric and adult oncology. NEXT CART goal is to develop new cell engineering approaches and treatments for R/R adult and pediatric neoplasms to evaluate in multicenter clinical trials. Here, we discuss the current limitations of T cell-based therapies and introduce our perspective on future developments. Advancement opportunities include developing allogeneic products, optimizing CAR signaling domains, combining cellular immunotherapies, multi-targeting strategies, and improving tumor-infiltrating lymphocytes (TILs)/T cell receptor (TCR) therapy. Furthermore, basic studies aim to identify novel tumor targets, tumor molecules in the tumor microenvironment that impact CAR efficacy, and strategies to enhance the efficiency of the immunological synapse between immune and tumor cells. Our perspective of current cellular immunotherapy underscores the potential of these treatments while acknowledging the existing hurdles that demand innovative solutions to develop their potential for cancer treatment fully.

## Introduction

In the last decade, CAR-T cell therapy has become 2^nd^ line of treatment in relapsed/refractory hematological malignancies. Currently, there are four approved CAR-T cell products directed to CD19 (CART-19) for B cell malignancies (axi-cel, tisa-cel, liso-cel, and brexu-cel) and two products targeting B-cell maturation antigen (BCMA) (CART-BCMA) (ide-cel and cilta-cel) for multiple myeloma (MM) patients (1, 2). Despite this growing number of CAR-T cell products approved for hematological malignancies, no CAR-T cell-based treatments are available to treat solid tumors, evidencing distinct limitations that guide new lines of research to develop improved CAR-T cells. Clinical studies have demonstrated that limitations in the CAR treatment differ depending on the CAR and the malignancy and could arise at different stages of the treatment.

Among the most common complications in CAR-T therapy are failures in producing autologous CAR-T cells, loss of target antigen, the barriers imposed by the tumor microenvironment (TME), and the lack of efficacy and persistence of CAR-T cells. Of interest, the immunological synapse (IS) constitutes a platform that involves changes in the cortical F-actin to transfer cytotoxic proteins from immune T and B cells to tumor cells, and has been widely studied in physiological conditions (3). The IS between CAR-T cells and tumor cells is not entirely understood and research in this field might provide new insights and targets to tackle some of these problems. Here, we outline the ongoing strategies developed to avoid events that decrease the efficacy of CAR-T treatment. As a Consortium, we will provide our perspective on how these strategies will evolve in the coming years to solve the main current limitations of these therapies.

## Off-the-shelf CAR therapies: developing alternatives to circumvent the failure of autologous CAR-T cell production

Tumor patients are often immunosuppressed and around 10% of patients enrolled in CAR-T therapy do not receive the CAR treatment due to a failure to produce autologous CAR-T cells (4, 5). The infusion of allogeneic universal CAR-T cells is a field in development to circumvent this problem. Current techniques knock out the T cell receptor α constant (TRAC) and CD52 genes/loci to reduce the risk of graft-versus-host disease (GvHD) and to avoid a host-versus-graft reaction, respectively. This option has been tested for CART-19 (6) and CART-BCMA cells (7), showing manageable safety. However, responses are still lower than those achieved with autologous CAR-T cells (5, 8). The previous depletion of the CD45RA^+^ fraction in CAR-T cells is another option to obtain a less alloreactive product enriched in memory T cells (9, 10). Indeed, our Consortium is administering allogenic memory CART-NKG2D cells in sarcoma patients (NCT06087341).

Other alternatives to circumvent the failure of autologous CAR-T cell production include allogeneic CAR-NK cells, which do not cause GVHD (11). Clinical trials have confirmed their safety (11), emerging as an alternative to producing allogeneic CAR cells (12). They are obtained from different sources, including cord blood (12), allogeneic induced pluripotent stem cells (iPSCs) (13), and peripheral blood. Despite the early expectations in this field, the intrinsic short half-life of NK cells might be responsible for the lower efficacy observed compared to CAR-T cells (14). Engineering CAR-NK cells with intrinsic NK receptor domains might improve their persistence. Indeed, various novel constructs that replace co-stimulatory and transmembrane domains of the CAR with more typical NK cell domains are being developed. Thus, NKG2D transmembrane, 2B4 co-stimulatory or DAP12 signaling domains (15) confer higher efficacy than CAR-NK cells containing CD28, 4-1BB, and CD3ζ; T cell signaling domains (16). Moreover, including specific cytokines in the construct should prolong CAR-NK cell persistence (17).

In addition, NK cells present additional advantages, such as CD16 expression that activates their antibody-dependent cell-mediated cytotoxicity (ADCC). Thus, combining CAR-NK cells with antibodies targeting tumor-associated antigens (TAAs) that bind to CD16 will trigger ADCC and could enhance their efficacy. Last but not least, CAR-NK treatment could also be combined with antibodies that block NK inhibitory receptors, such as NKG2A (18).

Another approach to explore in the clinic is exploiting the essential property of NK cells for activating other immune cells (15), including T cells and CAR-T cells (19). Indeed, a combined treatment based on CAR-T and NK cells enhances *in vivo* CAR-T efficacy (19), offering a therapeutic advantage.

## Optimization of CAR domains to enhance the IS formation and function

**Figure 1:**
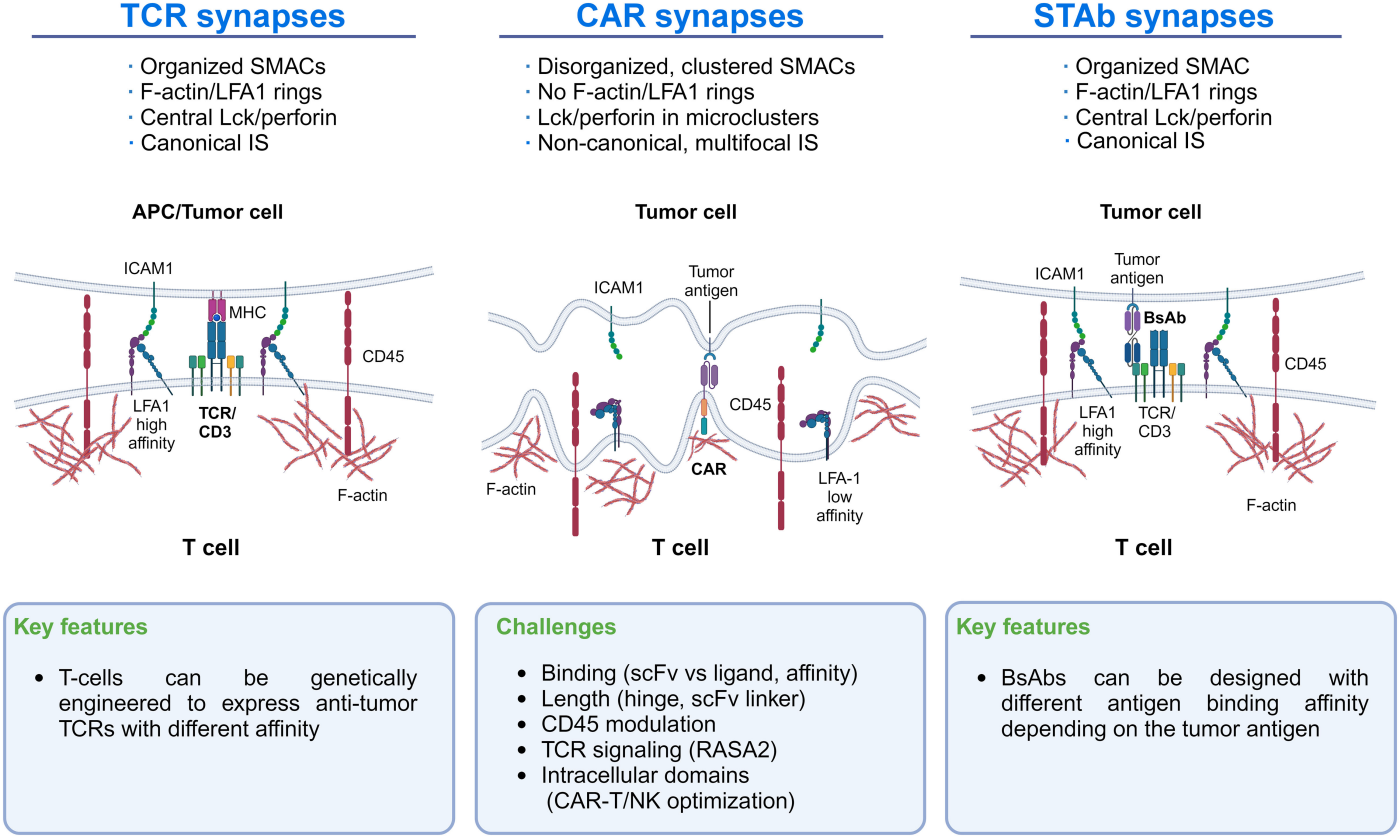
Immunological synapse features of TCRs, CAR-T cells, and STAb-T cells: The main differences in the IS of TCRs, CAR-T cells, and STAb-T cells are shown. These differences could guide new lines of research to improve CAR-T cells’ IS formation.

Research on CAR domain modification to optimize IS formation and function between CAR cells and their tumor cell targets represents a field in development that might improve clinical results. Indeed, CAR-T cells’ IS differs from that of TCRs and T cells secreting bispecific T-cell engager antibodies (STAb-T cells) (20). CAR-T cells’ IS relies mainly on the CAR interaction with the tumor antigen, which limits the stability and strength of the IS. In contrast, STAb-T cells recapitulate key features of the TCR-mediated IS more closely (see below). The key characteristics of the distinct IS are shown in **Figure 1** to illustrate differences that could guide new research lines to improve CAR-T cells’ IS formation and effector function.

Increasing LFA-1/ICAM-1 interaction around CAR-T cell IS, as occurs with TCR-mediated IS, might improve the efficacy of CAR-T therapy. Indeed, recent studies highlight the significance of LFA-1 expression in forming the IS in T and NK cells (21, 22) and the importance of the LFA-1/ICAM-1 axis in CAR-T cell/tumor cell interactions in solid tumors (23, 24) (**Figure 1**). Initially, CAR-T cell activation strongly depends on the expression level of ICAM-1 on tumor cells (24), where CAR-T cells detach from malignant cells more quickly than normal T cells due to LFA-1 downregulation in the IS (25). Therefore, improving LFA-1/ICAM-1 interaction in the CAR-T synapse might help to establish more efficient IS, which could be achieved by enforcing in the CAR-T the production of cytokines that upregulate cell adhesion molecules (see below). Another suitable approach could be to express an anti-ICAM-1 scFv on the CAR-T that would interact with ICAM-1 in the tumor cell. However, limitations of this strategy would be the difficulty of restricting the scFv-ICAM-1 interaction to the IS and whether the strength and stability of this interaction would be counterproductive for CAR-T cell function. Other critical factors that modulate the IS formation and CAR-T activity include scFv/ligand conformation, the hinge length, and the presence of additional CAR domains (26, 27). For instance, in 4-1BB CARs targeting CD22, shortening the linker of the scFv drives antigen-independent signaling. This tonic signaling enhanced IS formation and conferred superior effector function, a finding not observed in CD28-based CARs, suggesting that shorter linkers may benefit 4-1BB CARs (28).

Furthermore, adding an intracellular scaffolding protein binding site, such as PDZ domain, enhances the IS formation and improves CAR-T and CAR-NK efficacy against solid tumors (29). Also, blocking TCR-signaling inhibitors, such as RASA2, improves the IS function, providing larger synaptic areas, increased lytic granules, and pZAP70 accumulation at the IS (30).

Studies analyzing CART-19 interactions have raised a “size exclusion” model whereby CAR-T activation depends on the size difference between the CAR-antigen pair and CD45 on the membrane. Precisely, CD19 engagement with CART-19 results in a narrow intermembrane space that excludes CD45, favoring CAR phosphorylation and CAR-T cell activation. Increasing the size of CAR extracellular domains attenuates CART-19 cell activation, and increasing the size of CD45 enhances CART-19 activation (31).

Several ex-vivo approaches based on artificial intelligence analyses of F-actin reorganization at the IS have assessed the IS quality made by CAR-T cells (32, 33). Indeed, *in vitro* learning-based IS quality based on F-actin measurements correlates with patient clinical outcomes upon CAR-T therapy (34). This strategy could provide guidelines for designing and optimizing CAR constructs and also optimize T cell-redirecting strategies such as STAb-T cells (see below) for potential clinical developments.

## Improving CAR recognition domains to avoid early CAR-T disappearance

Most CARs used in the clinic are derived from murine single-chain variable fragments (scFvs), which induce the development of human anti-murine antibodies (HAMAs) by the patients, leading to early CAR-T cell disappearance, without time to achieve responses in the patient (35). Current strategies to avoid this event include humanizing murine CARs (5). Also, camelid heavy-chain variable domains (VHHs) present higher homology to human variable domains, being less immunogenic than murine scFv (2). These two options have shown higher rates of initial responses in MM than those derived from murine scFv (1, 2, 5). In addition, newer CAR constructs contain synthetic antigen-binding domains that are fully human, and have shown outstanding results, representing a promising alternative (36).

## Multi-targeting to avoid relapses due to target antigen loss, tumor heterogeneity, and on-target, off-tumor toxicities

CART-19 therapy in pediatric B-ALL represents a curative option, with an overall remission rate of 82% after 38.8 months, and a median overall survival not reached (8). However, relapses still occur due to a target antigen loss on tumor cells. It happens more frequently in pediatric ALL patients (7) than in adult patients (13), with 68% vs. 13% of relapsed patients after CART-19, respectively. Moreover, some antigens are more prone to disappear than others. Thus, loss of BCMA in MM after CAR-T treatment is hardly observed or only suspected (5), whereas target antigen loss in solid tumors has been observed (37).

Multi-targeting CAR-T strategies, such as CARs in tandem/dual, bicistronic formats or co-administration/co-transduction and sequential approaches targeting CD19/CD22 or CD19/CD20, have been used in clinical studies in B-ALL as an alternative to avoid these relapses (38–43). Although results are encouraging, this strategy is hampered by the loss of the second target antigen (44), and, therefore, other approaches are being developed, such as CARs directed to antigens that do not undergo loss of expression or tri-specific approaches combining CD19/CD20/CD22 (45).

In other pediatric malignancies such as acute myeloid leukemia (AML), neuroblastoma, hepatoblastoma, or osteosarcoma, CAR-T cell therapy is still inefficient due to the heterogeneity of the tumor (46). Thus, in AML, dual/tandem CAR approaches targeting multiple antigens, including CD123/CLL1 (30), CD123/CD33, or CD33/CLL1/CD123 are being tested to fight tumor heterogeneity (47). In pediatric osteosarcoma, newer CAR designs, such as switchable CARs, are being evaluated in our Consortium (48) to solve tumor heterogeneity.

Moreover, finding tumor-specific antigens to avoid toxicities remains challenging for solid tumors. Dual gate-logic CARs, which require recognition of two antigens to become activated (49), and split, universal, and programmable (SUPRA) CARs (50) will help reduce on-target/off-tumor toxicities.

## Shortening of CAR production and other approaches to avoid immunosenescence and improve CAR persistence

The most frequent relapses after achieving a response occur due to a lack of CAR-T cell persistence. The age of the patient (51), the number of previous lines of treatments (52), and the production of CAR-T cells (53) induce terminal T cell differentiation, where T cells become immunosenescent, presenting a sorter half-life and, therefore, a lower persistence in the patient. Indeed, MM patients, an elderly population, achieve 100% of objective responses after CAR-T treatment; however, a proportion of them end up relapsing before the 1^st^ year with a lack of CAR-T cell persistence (5). Conversely, pediatric and young ALL patients treated with CAR-T cells have not relapsed after 38.8 months and still present B-cell aplasia, indicating CAR-T cell persistence (8). Different production techniques that shorten the time of manufacturing or adding drugs to avoid CAR-T cell differentiation (54) will be helpful strategies to prevent this issue.

## TRUCKs to improve the quality of the IS and to fight the TME barriers

Additional factors that impact CAR-T persistence and efficacy are the barriers of the TME. Indeed, CAR-T cells can become anergic when they arrive at the TME. Cell-cell communication between tumor and CAR-T cells through the IS might lead to these events. It is well-known that the IS serves as a platform to transfer cytotoxic immune and tumor proteins that may impact the activity of immune cells. Indeed, the quality of the IS dictates the anti-tumor efficacy of T cells, NK cells, and CAR-T cells (33). Thus, an algorithm that measures the quality of the IS based on four parameters (F-actin, perforin, tumor antigen, and pZeta) demonstrated that the CAR-T IS correlates with the clinical response in patients treated with CART-Kappa cells (NCT00881920), supporting that this “ex vivo” synaptic parameter can predict the clinical response to CAR treatment (34).

Developing the so-called fourth-generation CAR-T cells or TRUCKs (T cells redirected for universal cytokine-mediated killing) could serve as a strategy to use molecules that stabilize and/or potentiate the IS between malignant and TRUCK-like cells. TRUCKs require CAR-T transduction with a transgene payload encoding various genes that may improve CAR-T efficacy in the TME (55, 56). In this regard, TME signals modulate the expression of some adhesion/costimulatory molecules that affect the quality of the CAR-T IS (57). In this regard, IFNγ produced by CAR-T cells upregulates ICAM-1 on tumor cells and strengthens CAR-T and tumor cell interaction, facilitating tumor killing in solid tumors (24, 58). Therefore, gene payloads encoding genes promoting cell adhesion, i.e. inducing the expression of LFA-1 (αLβ2) or other relevant integrins in the CAR-T cell, could be a valuable strategy to strengthen the IS of tumor cells and CAR-T cells.

In this line, our Consortium is evaluating the combination of CAR-T cells with other approaches targeting IFNγ and other cytokines to promote CAR-T/tumor cell IS. Indeed, radiotherapy, which increases IFNγ on the TME (59), or armed oncolytic viruses producing CXCR3 ligands, such as CXCL10, enhancing the avidity of LFA-1 on CAR-T cells (60), are being investigated by us.

Moreover, to fight the TME barriers, novel TRUCKs that secrete different molecules are approaches under investigation (61). The TRUCK concept is also suitable for other cells susceptible to carrying a CAR, such as NK cells and macrophages (62). New approaches include the use of payloads of a variety of immunomodulatory genes besides cytokines (63). For example, IL12 has been the subject of several studies addressing this issue, using distinct strategies to express IL12 in TILs or tumors (64, 65). Despite being a promising strategy, it has become evident that there is a need to adapt these strategies to the particularities of each TME. Thus, our Consortium is developing TRUCKs expressing transgenes under the control of promoters explicitly activated in the TME of each type of tumor. Identifying promoters activated by metabolites or signals overrepresented and/or specific to particular TMEs is crucial in restricting the activation of the gene payload to the tumor, minimizing toxicities.

## TILs and TCRs to fight the heterogeneity of solid tumors

Our Consortium is also developing TILs, a group of lymphocytes that include T cells, NK cells, and other innate lymphocytes that infiltrate tumors and recognize and destroy tumor cells. The main advantages of TILs include their polyclonality, which enables targeting multiple tumor antigens and circumvents the problem of antigen heterogeneity in solid tumors. Moreover, by being isolated from tumors, they are already equipped with chemokine receptors, providing a higher capacity for homing into the tumor. Finally, most TILs present a TCR with high affinity to TAAs and neoantigens, which decreases the possible on-target, off-tumor toxicities. T cell activation is mediated through highly organized and dynamic IS where the TCRs and MHC-peptide complexes interact (66–68).

TIL therapy was first described as an adoptive cell therapy by Steven A. Rosenberg in 1988 (69), where they isolated and expanded TILs and re-infused them into patients with metastatic malignant melanoma, causing tumor regression. Afterwards, in 2015, they treated nine patients with metastatic cervical cancer, obtaining 33% of durable objective responses after a single infusion of TILs (NCT01585428) (70).

Despite their potential, TIL therapy still faces several challenges. The main one is the current procedure to obtain TILs, which requires two months of *in vitro* expansion to get the necessary number of TILs to treat patients. This long period may lead to a product with poor persistence (71). In addition, some patients with rapidly progressing diseases cannot wait long for the isolated TILs to expand. Recent strategies to improve TIL persistence and efficacy include the knockout of endogenous TCR (72) and PD1 to avoid T cell exhaustion and overcome the particularly immunosuppressive TME of solid tumors.

Another limitation is that not all tumors are suitable for isolating active TILs, and specific TAAs are lacking. Engineered TCR-based therapies have emerged to address this issue (73–76). This therapy arose with the discovery that TILs from different patients recognize the same antigens expressed in tumor cells, such as MART-1 and glycoprotein 100 (gp100) (77). Engineered TCRs require T cell transduction with antigen-specific TCRα and β chains to generate tumor-specific T cells, expansion, and reinfusion in the patient (76). They preserve many advantages inherent to natural TCRs, such as fully mediating TCR signaling. Moreover, TCRs are sensitive to small epitope densities (67, 78), and their activation depends on the antigen presented by the MHC of a large target antigen pool (79), which allows modulating TCR affinity, especially against low-density targets (80).

In January 2022, the FDA approved the first TCR-based therapeutic for treating uveal melanoma targeting gp100 presented on HLA-A2 (81). In February 2024, Iovance Biotherapeutics announced the FDA approval of AMTAGVI™ (lifileucel), a tumor-derived autologous T-cell immunotherapy for treating adult patients with unresectable or metastatic melanoma (82). Next, in 2024, the FDA is expected to approve an engineered TCR T cell therapy targeting MAGE-A4 to treat synovial sarcoma (83).

## STAb-T cells, an optimal tool to enhance the efficacy of adoptive T cell therapy

An attractive and emerging approach in adoptive T cell therapy involves the infusion of T cells genetically modified to secrete T cell-redirecting bispecific Antibodies (STAb-T cells) directed to CD3 and to a tumor-associated antigen (84). Therefore, STAb-T strategy combines aspects of antibody- and cell-based therapies and might solve some of the limitations of the T cell-redirecting strategies currently used in the clinic. Indeed, unlike systemically administered bispecific antibodies (bsAbs), STAb-T cells have the ability to migrate to areas of inflammation and damage (as can be tumors) and actively extravasate to tissues by crossing the vascular-endothelial barrier. They also have a long half-life, and produce a constant release of the therapeutic bsAb, which eliminates the problems associated with the manufacture, storage, and administration of antibodies (84). Importantly, contrary to the membrane-anchored CAR, the soluble bsAbs secreted by STAb-T cells can recruit the entire pool of T lymphocytes, both engineered and unmodified bystander T cells, present at the tumor site (85–87), enhancing the anti-tumor response.

Moreover, when CAR-T cells encounter tumor cells, they form a disorganized synapse differing from the canonical “bull’s eye” structure initiated by TCR interactions with the MHC-peptide complex (25, 66, 85, 88). Unlike CARs, small-sized T cell-engaging bsAbs induce the formation of a seemingly canonical IS between T lymphocytes and tumor cells (**Figure 1**). Proper synapse formation plays an essential role in the T cell activation process triggered by the TCR and in the polarized secretion of lytic granules or cytokines to the synaptic cleft (89–91). Further studies will be necessary to determine whether such differences could represent an advantage of STAb-T cells over CAR-T cells in cancer immunotherapy. Recently, studies have demonstrated a higher efficacy of STAb-T cells vs. CAR-T cells targeting the same antigen in animal models of different hematological tumors (89–91). On the other hand, the generation of CAR-T cells that secrete bsAbs simultaneously with CAR expression (CAR-STAb-T cells) (92–95) and the generation of STAb-T cells (94) are attracting much interest. Our Consortium is leading research lines in these areas.

## Adoptive T cell therapy to treat other diseases

Finally, all the strategies discussed above also offer a high potential to treat other pathologies than cancer, including autoimmune and infectious diseases (96). CART-19 therapy has already reached the clinic, with ongoing trials to analyze the safety and efficacy of CART-19 cells in treating systemic lupus erythematosus (97) (NCT05030779; NCT03030976). Other approaches for targeting autoreactive B cells are being developed (98, 99).

Regarding infectious diseases, opportunistic infections affecting immunocompromised patients remain a significant cause of morbidity and mortality (100). Since their immune system is impaired, adoptive T cell therapy in these patients would depend on allogeneic T cells. In this sense, our Consortium has conducted a study evaluating the treatment of immunocompromised patients suffering from viral or fungal infections with allogeneic CD45RA-T lymphocytes from healthy donors containing pathogen-specific memory T cells (101). Moreover, this procedure is being tested in a clinical trial against SARS-CoV-2 (NCT04578210) with promising results (102).

## Concluding remarks

Adoptive cell-based immunotherapy has an enormous potential to treat refractory tumors and other diseases. In the coming years, we expect all the mentioned strategies will enable therapy optimization, especially for solid tumors and other diseases. Bringing together the efforts of basic/translational and clinical researchers will help to achieve this. To this end, our Consortium points at these hurdles that demand innovative solutions to fulfill their full potential in cancer therapy.

## Data availability statement

The original contributions presented in the study are included in the article/supplementary material. Further inquiries can be directed to the corresponding authors.

## Author contributions

BM-A: Conceptualization, Funding acquisition, Writing – original draft, Writing – review & editing. BB: Conceptualization, Funding acquisition, Writing – original draft, Writing – review & editing. ÁG-M: Conceptualization, Funding acquisition, Writing – original draft, Writing – review & editing. LH: Conceptualization, Funding acquisition, Writing – original draft, Writing – review & editing. JM: Conceptualization, Funding acquisition, Writing – original draft, Writing – review & editing. GP-C: Conceptualization, Funding acquisition, Writing – original draft, Writing – review & editing. NC: Writing – original draft, Writing – review & editing.

## Group members of ‘Next Generation CART MAD Consortium’

Hospital Universitario la Paz, Universidad Autonoma de Madrid: Jordi Minguillón, Lucía Fernández, Halin Bareke, Andrés París-Muñoz, Adriana Mañas, Cristina Aguirre-Portolés, Alfonso Navarro-Zapata, Carmen Mestre-Durán, Marta Ibáñez-Navarro, Laura Clares-Villa, Sara Naharro and Antonio Perez-Martinez (PI),

Consejo Superior de Investigaciones Científicas (CSIC): Marcos Aldea, María Gaibar, Javier Ruiz-Navarro, Manuel Izquierdo and Juan M. Zapata (PI).

Fundación Jimenez Díaz: Esperanza Esquinas Tarifa, Juana Serrano-López, Pilar Llamas-Sillero and Beatriz Martin-Antonio (PI).

Hospital Universitario Infantil Niño Jesus: África González Murillo, Elena García Sánchez, Jorge García Martínez, Alba Rubio San Simón, Blanca Herrero, Gonzalo García Aguilera, Beatriz Horcajo Morera and Manuel Ramírez Orellana (PI).

Hospital Universitario 12 de Octubre: Belén Blanco-Durango, Anais Jiménez-Reinoso, Rodrigo Lázaro-Gorines, Ivana Zagorac, Antonio Tapia Galisteo, Ángel Ramirez-Fernández, Laura Díez-Alonso, Carmen Dominguez-Alonso, Ainhoa Erce-Llamazares, Oana Hangiu, Laura Rubio Pérez, Marina Gómez Rosel, Alejandro Segura Tudela, Javier Arroyo-Ródena and Luis Álvarez-Vallina (PI).

Instituto de Salud Carlos III (ISCIII): Miguel Ángel Rodríguez Milla, Patricia García Rodríguez, Beatriz Somovilla Crespo and Javier García-Castro (PI).
